# Sleep Deprivation and Neurological Disorders

**DOI:** 10.1155/2020/5764017

**Published:** 2020-11-23

**Authors:** Muhammed Bishir, Abid Bhat, Musthafa Mohamed Essa, Okobi Ekpo, Amadi O. Ihunwo, Vishnu Priya Veeraraghavan, Surapaneni Krishna Mohan, Arehally M. Mahalakshmi, Bipul Ray, Sunanda Tuladhar, Sulie Chang, Saravana Babu Chidambaram, Meena Kishore Sakharkar, Gilles J. Guillemin, M. Walid Qoronfleh, David M. Ojcius

**Affiliations:** ^1^Department of Pharmacology, JSS College of Pharmacy, JSS Academy of Higher Education & Research, Mysuru, India; ^2^Department of Food Science and Nutrition, CAMS, Sultan Qaboos University, Muscat, Oman; ^3^Ageing and Dementia Research Group, Sultan Qaboos University, Muscat, Oman; ^4^Department of Medical Bioscience, Faculty of Natural Science, University of the Western Cape, Robert Sobukwe Road, Bellville 7535, South Africa; ^5^School of Anatomical Sciences, Faculty of Health Sciences, University of the Witwatersrand, Johannesburg, York Road, Parktown, 2193 Johannesburg, South Africa; ^6^Department of Biochemistry, Saveetha Dental College, Saveetha Institute of Medical and Technical Sciences, Saveetha University, 600 077, Chennai, India; ^7^Department of Biochemistry, Panimalar Medical College Hospital & Research Institute, Varadharajapuram, Poonamallee, 600123, Chennai, India; ^8^Department of Biological Sciences and Institute of Neuroimmune Pharmacology, Seton Hall University, 400 South Orange Ave South Orange, NJ 07079, USA; ^9^College of Pharmacy and Nutrition, University of Saskatchewan, 107, Wiggins Road, Saskatoon, SK, Canada S7N 5C9; ^10^Neuroinflammation Group, Faculty of Medicine and Health Sciences, Macquarie University, NSW 2109, Australia; ^11^Research & Policy Department, World Innovation Summit for Health (WISH), Qatar Foundation, P.O. Box 5825, Doha, Qatar; ^12^Department of Biomedical Sciences, University of the Pacific, Arthur Dugoni School of Dentistry, San Francisco, CA, USA

## Abstract

Sleep plays an important role in maintaining neuronal circuitry, signalling and helps maintain overall health and wellbeing. Sleep deprivation (SD) disturbs the circadian physiology and exerts a negative impact on brain and behavioural functions. SD impairs the cellular clearance of misfolded neurotoxin proteins like *α*-synuclein, amyloid-*β*, and tau which are involved in major neurodegenerative diseases like Alzheimer's disease and Parkinson's disease. In addition, SD is also shown to affect the glymphatic system, a glial-dependent metabolic waste clearance pathway, causing accumulation of misfolded faulty proteins in synaptic compartments resulting in cognitive decline. Also, SD affects the immunological and redox system resulting in neuroinflammation and oxidative stress. Hence, it is important to understand the molecular and biochemical alterations that are the causative factors leading to these pathophysiological effects on the neuronal system. This review is an attempt in this direction. It provides up-to-date information on the alterations in the key processes, pathways, and proteins that are negatively affected by SD and become reasons for neurological disorders over a prolonged period of time, if left unattended.

## 1. Introduction

Sleep is a ubiquitous phenomenon occurring in life forms of the animal kingdom and has been shown to be present from Drosophila melanogaster (fruit fly) to human beings. Sleep is a vital component for healthy brain function, and sleep deprivation (SD) is the reduction in sleep time below an individual's baseline requirement while sleep restriction (SR) refers to partial loss of sleep. SD and SR have been reported to affect overall wellness and health, including, but not limited to lowering in the immune system, decrease in cognitive function and memory, learning, and disruption in emotional wellbeing [1]. National Sleep Foundation, USA, suggests that 7–8 h of sleep is essential for maintenance and restoration of metabolic homeostasis [2]. There are two stages of sleep: (i) nonrapid eye movement (NREM) and (ii) rapid eye movement (REM). NREM is subdivided into four different stages based on the depth and wave patterns, movement of the eye, and muscle strength during sleep. REM sleep is characterized by uneven brain wave activity, muscle atonia, and increased eyeball movements [3]. Sleep is regulated by two processes that work independently and influences sleep and sleep-related variables in conjunction “Rheostat” [4]: (1) Circadian rhythm—a process maintained by the biological clock in the suprachiasmatic nucleus (SCN) in the hypothalamus, which regulates sleep–wake cycles in response to the input from retina [5]; (2) homeostatic process—loss of sleep is compensated by extending subsequent sleep which is a function of waking duration and intermittent naps during the wake period. During wakeful hours, the tendency to sleep gradually increases with time and attains a critical threshold; this urge to sleep is referred to as homeostatic.

Sleep helps to maintain metabolic homeostasis through neural, hormonal, and immune supports [6]. NREM sleep is characterized by a low metabolic rate and an increase in brain temperature which helps to overcome the damages that are introduced during the wake cycle [7]. A study conducted on shift workers revealed SD alters glucose and lipid metabolism [8], which suggests the role of sleep in metabolic dysfunction. Krause et al. reported that SD affects attention and working memory, positive and negative emotions, and hippocampal learning [9].

The increasing number of people across the globe are being affected with the epidemic of SD [10]. SD impairs sympathetic functions which in turn causes metabolic dysregulation [11, 12]. Furthermore, SD modulates immune functions and increases the release of proinflammatory cytokines such as Interleukin 6 (IL-6), Tumour necrosis factor (TNF-*α*), and C-reactive protein (CRP) [13]. Sleep restriction increases prolactin and oxytocin and decreases body weight in pregnant rats. Offsprings from these rats show decreased hippocampal brain-derived neurotrophic factor (BDNF) expression, suggesting the detrimental role of SR on neuronal growth factors during the developmental stage [14]. Sleep loss during developmental stages reduces brain size and alters behaviour and neural homeostasis [15]. Electroencephalogram studies have revealed that SD promotes interictal epileptiform discharges and neuronal excitability causing activation of seizure episodes [16]. SD has also been associated with the impairment of cognitive function in humans. A recent study showed that one-night SD increased amyloid-*β* burden in the hippocampal region [17]. Thus, mounting evidences reveal that SD is linked with many neurodegenerative diseases and neurological disorders. In this review, we attempt to provide information on the links between sleep deprivation/restriction on the changes in gene expression and pathophysiological mechanism associated with neurological disorders.

## 2. Sleep and Brain Anatomical Structures

Sleep and its mechanism are controlled by defined regions in the brain. Microanatomically, cell bodies of neurons that produce neurotransmitters playing a role in sleep mechanisms are usually located in one region while the terminal ends of the neuronal axons project elsewhere [18]. In the mammalian brain, the cell bodies of the neurons involved in sleep are located in the brainstem while the axons end in centres located in the cerebral hemisphere. Sleep entails a patterned interaction between the cerebral cortex, thalamus, and subcortical areas like the brainstem. According to [19], “The ebb and flow of neurotransmitters switches our brains between sleep and wakefulness in carefully regulated cycles in several brain regions.”

The hypothalamus is located deep in the brain, proximal to the pituitary gland. It contains thousands of nerve cell bodies called the suprachiasmatic nuclei (SCN), which receive information about light exposure to control the sleep and arousal cycle [20]. The pineal gland lies in the depression between the superior colliculi. Through its numerous connections, the production of the sleep-promoting neurohormone melatonin is regulated; hence, it plays a key function in regulating the circadian rhythm. The amygdala, a structure known in the processing of emotions, has been suggested to be very active during rapid eye movement (REM) sleep, which explains the co-occurrence of mood disorders with sleep abnormalities [21].

Neurons of the reticular activating system are central to the regulation of wakefulness. The brain stem components (midbrain, pons, and medulla oblongata) have connections with the hypothalamus to control the wake and sleep cycle. The midbrain is associated with vision, hearing, motor control, sleep and wake cycles, alertness, and temperature regulation. The pons and medulla oblongata in particular have connections with descending neural pathways that maintain muscle activity and body posture and limb movements at the relaxed state. Brainstem nuclei that are involved in sleep processing include the cholinergic nuclei at the pons–midbrain junction, the raphe nuclei, tuberomammillary nuclei, and locus coeruleus [18]. The thalamus acts as a relay centre for information from the main sense organs to the cortex. It is very active during REM sleep as the ascending brainstem reticular activating system (ARAS) relays at multiple intermediary sites (including the thalamus), to activate the forebrain during waking and REM sleep [22]. Damage to the thalamus may affect proper brain function during both wakefulness and sleep in humans [23]. Cholinergic neurons in the basal forebrain region have been reported to promote sleep via the release of the cellular energy by-product, adenosine. Caffeine and some medications are known to counteract sleepiness by blocking the actions of adenosine [24].

## 3. Sleep and Neural Circuits

**Figure 1:**
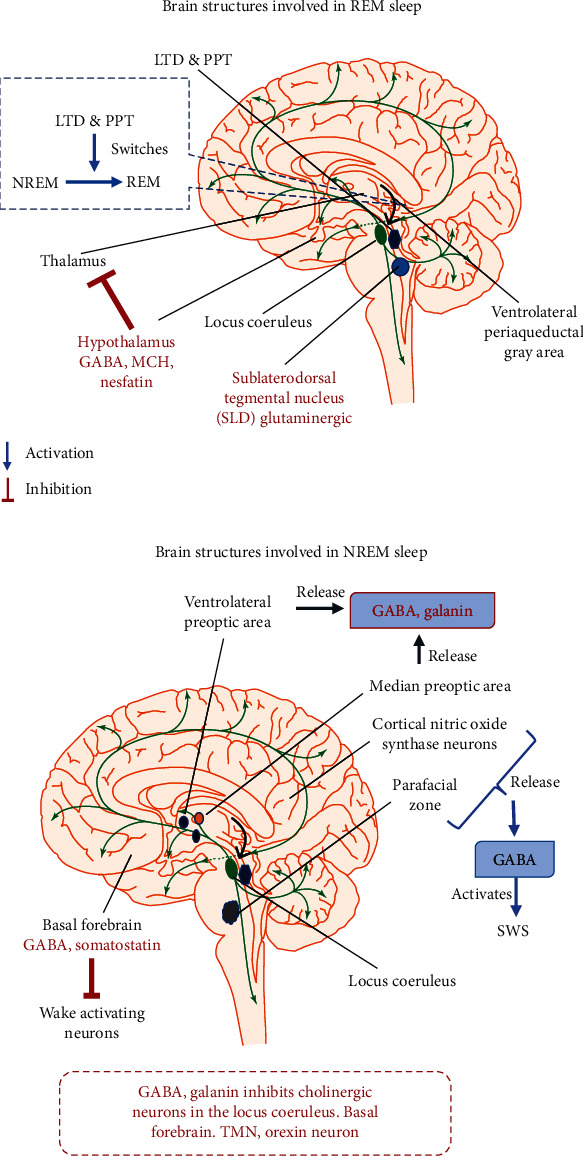
(a) Brain structures involved in REM sleep. PPT/LDT initiates firing during REM sleep and helps in switching between NREM and REM sleep. GABAergic neurons in the hypothalamus promote REM sleep by inactivating vlPAG/dDpMe REM-off GABAergic neurons. SLD consists of projections to glycinergic neuron Raphe magnus, ventral and alpha gigantocellular nuclei, lateral paragigantocellular nucleus, and also spinal, facial, trigeminal neurons and thereby produce muscle atonia during REM sleep. (b) Brain structures involved in NREM sleep. Ventrolateral preoptic area and median preoptic nucleus contain GABAergic neurons that release galanin which inhibit cholinergic neurons in regions like locus coeruleus, Basal Forebrain, TMN, and orexin neurons, thereby inhibiting arousal. The basal forebrain consists of GABAergic neurons that inhibit cortical activation and somatostatin inhibits wake active neurons in the basal forebrain. Parafacial zone releases GABA onto parabrachial neurons which in turn release glutamate onto cortically projecting neurons of the BF, hence promoting SWS. Cortical nitric oxide synthase neurons release GABA and promote SWS.

The involvement of neural circuits in sleep-wake cycles was first studied by Magoun and Moruzzi in the year 1949 [25]. They showed that stimulation of cholinergic neurons near pons and midbrain causes wakefulness and arousal. In addition, stimulation of the thalamic region with a low-frequency pulse produces a slow-wave sleep. This study provided an insight on the interaction between the thalamus and cortex during sleep (Figure 1).

### 3.1. Neural Circuits Involved in NREM Sleep

#### 3.1.1. Preoptic Area (POA)

POA is the rostral part of the hypothalamus and is mainly involved in the thermoregulation of the body. In 1968, McGinty and Sterman's research on cats and rats revealed that POA contains neurons that promote sleep [26]. They also showed that certain neurons in the POA and Basal Forebrain (BF) are active during REM and NREM sleep [27]. The analysis of the Fos expression revealed that the ventrolateral preoptic area (VLPO) and median preoptic nucleus (MnPO) consist of neurons essential for NREM sleep [28]. Lesions in the VLPO and MnPO produced a long-lasting decrease in sleep [29]. Furthermore, these neurons are GABAergic. Interestingly, VLPO neurons release a neuropeptide called “galanin,” which inhibits cholinergic neurons of the locus coeruleus, Basal Forebrain, TMN, orexin neurons, and causes sleep arousal [30].

#### 3.1.2. Basal Forebrain (BF)

BF is innervated by cholinergic neurons which govern cortical activation during wakefulness. The inhibition of cortical activation is governed by the GABAergic neurons and promotes slow-wave sleep. GABAergic neurons initiate firing at NREM onset and have their maximal output throughout the NREM cycle [31]. BF neurons produce somatostatin and are active during NREM sleep, and optogenetic stimulation of these neurons is shown to increase NREM sleep [32]. Somatostatin promotes NREM sleep by inhibiting wake active neurons in mice [32, 33]. On the other hand, prolonged wakefulness increases GABA_A_ receptors in BF cholinergic neurons, and the GABA-mediated inhibition of cholinergic neurons decreases the cortical activity [34].

#### 3.1.3. Parafacial Zone (PZ)

Caudal brainstem neurons promote NREM sleep [35]. Most of the neurons in the parafacial zone are GABAergic and glycinergic. They express Fos during sleep, and lesions of these neurons are shown to increase wakefulness. PZ neurons release GABA onto parabrachial neurons which in turn release glutamate onto cortically projecting neurons of the BF thereby potentiating slow-wave sleep and regulate cortical EEG [36]. Chemogenetic inhibition of PZ neurons markedly declines NREM sleep even after SD, indicating that PZ neurons play key roles in NREM sleep [37].

#### 3.1.4. Cortical Neuronal Nitric Oxide Synthase (nNOS)

Nitric oxide synthase neurons regulate sleep homeostasis and cortical rhythm through the release of GABA and nitric oxide (NO). Interestingly, the nNOS knockout mice showed a decrease in NREM sleep [38], which indicates the major role of nNOS in NREM. Majority of the neurons present in the cortical regions are wake-promoting and are GABAergic neurons, wherein the slow-wave activity during the NREM sleep directly correlates with the expression of nNOS [39].

### 3.2. Neural Circuits Involved in REM Sleep

Evidence from recent studies suggests that GABAergic/glycinergic neurons in the medullary reticular formation release melatonin, and GABAergic neurons present in the hypothalamus regulate REM sleep. Pedunculopontine and Laterodorsal Tegmental Nuclei**—**cholinergic LDT/PPT neurons and glutaminergic neurons of the pons play a crucial role in regulating REM sleep. These reports suggest the existence of a strong neural circuit in the regulation of REM sleep

#### 3.2.1. Pedunculopontine and Laterodorsal Tegmental Nuclei (PPT/LDT)

The administration of carbachol, a cholinergic agonist, by intracerebroventricular injection into the laterodorsal region of the pons produces a high degree of REM sleep in cats and rodents, indicating that acetylcholine levels are high during sleep [40]. These neurons initiate firing during the beginning of REM sleep, suggesting that they help in switching between REM and NREM sleep [41].

#### 3.2.2. GABAergic Neurons in the Hypothalamus

An increased number of c-Fos immunopositive cells were recorded in various regions of the hypothalamus including zona increta, perifornical area, and lateral hypothalamic area following REM sleep [42]. GABAergic neurons present in this region are immunoreactive to melanin-concentrating hormone (MCH) and nesfatin both of which regulate REM sleep [42, 43]. The optogenetic activation of MCH neurons facilitates the switching between NREM and REM sleep and also increases the duration of REM sleep [43, 44]. The inhibition of lateral hypothalamic (LH) neurons with muscimol, a GABA_A_ receptor agonist, is shown to completely inhibit REM sleep and increase the NREM sleep episodes. The bilateral administration of muscimol increases the expression of cFOS^+^/GABA^+^ and GABA^+^ in vlPAG and dorsal deep mesencephalic reticular nucleus (dDpMe) regions [45]. These results indicate that MCH^+^/GABA^+^ and MCH^−^/GABA^+^ neurons in the LH promote REM sleep by the inactivation of vlPAG/dDpMe neurons.

#### 3.2.3. Sublaterodorsal Nucleus (SLD) Region

Chemical lesions and optogenetic stimulation studies have shown that SLD contains neurons responsible for the initiation and maintenance of REM sleep [46]. In addition to cFO expression following REM sleep, the neurons in SLD also show the existence of glutamatergic markers, which reveal that REM sleep-promoting neurons in SLD are glutaminergic in nature [47]. SLD sends direct efferent projections to glycinergic neurons in the Raphe magnus, ventral and alpha gigantocellular nuclei, lateral paragigantocellular nucleus, and also spinal, facial, trigeminal neurons [48, 49]. On the other hand, the ventromedial medulla-glycinergic/GABAergic is populated near rostral to inferior olive in the ventral gigantocellular reticular (GiV) and the alpha gigantocellular reticular (GiA) nuclei [45]. These neurons fire very fast in REM, slower in NREM, and moderately in the wake cycle, indicating their role in muscle atonia [50], and lesions to these neurons are shown to disrupt atonia of REM sleep [51]. Another study proposes that neurons in SLD directly trigger spinal interneurons through glycinergic/GABAergic components, which further supports their role in muscle atonia [52].

#### 3.2.4. Medullary Reticular Formation

GABAergic and glycinergic neurons regulate REM sleep. They receive input signals from SLD and innervate the motor neurons of the brainstem and spinal motor neurons. Upon stimulation, they produce glycinergic Inhibitory Postsynaptic Potential (IPSP) in motor neurons [49]. Neurons of the dorsal paragigantocellular reticular (DPGi) and lateral paragigantocellular (LPGi) promote REM sleep by inhibiting REM sleep suppressing neurons of the pons like LC, DRN, and vlPAG/LPT [53]. Photostimulation of neurons in LPGi and vlPAG promotes REM sleep, and inhibition of these neurons produces opposite effects [54]. These findings suggest that medullary neurons not only promote atonia but are also involved in REM sleep regulation. Further studies are required to understand whether REM sleep is driven by the medulla or pons or both the regions of the brain.

## 4. Genomics and Sleep Deprivation

Hippocampus is the key brain structure involved in spatial, contextual, and declarative memory. The formation of memory depends upon the expression of various genes/proteins [55, 56]. Hippocampal dependent memory consolidation is critically affected in SD [57, 58]. SD is shown to affect the signalling mechanisms that regulate transcription and translation processes involved in memory [59]. A genome-wide microarray analysis by Vecsey et al. [60] showed the group of genes that are differentially regulated in SD animals in comparison to animals that have normal sleep patterns. *Tsc22d3*, *Prkab2*, *Adamts2*, *Htr1a*, *Kcnv1*, and *Sirt7* are the genes that show upregulation*. Tsc22d3* negatively regulates memory consolidation and neural plasticity, and its expression is reported to be upregulated after SD. *Prkab2*, a subunit of AMPK, undergoes hyperphosphorylation following SD and alters homeostatic response. In addition, the expression of certain genes in the cortex such as *Arc/Arg3.1*, *Fos*, *Hnrpdl*, *Rbm3*, and the chaperones *Hspa5/Bip* and *Hspa8* is also altered by SD [60].

SD impairs protein synthesis; it downregulates the genes like *Eif4e2* and *Eif5*, which are involved in the initiation of transcription and those genes like *Rprd2*, *Rbm3*, *Hnrpdl*, *Cirbp*, *RbmX*, and *Denr* involved in the process of translation [60]. Please refer to the supplementary materials (available here) for the table and graphical presentation of genes up- and downregulated following SD.

## 5. Sleep and Neurological Disorders

### 5.1. Alzheimer's Disease

Alzheimer's disease (AD) is a progressive neurodegenerative disease pathologically characterized by the deposition of extracellular amyloid *β*- (A*β*-) plaques, intracellular tangles, and neuronal loss [61]. Studies have shown a direct correlation between SD and neuropathological events associated with AD [62]. Sleep plays a crucial role in clearing the toxic metabolites produced in the brain. Two investigations using photon microscopy have shown that amyloid *β* in the brain is cleared by astroglial-mediated interstitial fluid (ISF) bulk flow called “Glymphatic Pathway” and also by *γ*-oscillations during REM sleep [63]. A*β* clearance is increased by 25-30% during sleep in comparison to wake state [64]. Sleep enhances interstitial fluid (ISF) to cerebrospinal fluid (CSF) bulk flow, thereby increasing the A*β* clearance [65]. On the other hand, SD increases the A*β* burden and its clearance leads to AD [65, 66]. Clinical reports indicate that the levels of A*β* in CSF are high before sleep and low after sleep [67]. Positron emission tomography (PET) analyses show that SD increases A*β* in the hippocampus, precuneus, thalamus, and cortex of human subjects [17]. This deposition of A*β* brings structural and functional changes in AD brains. The aggregation of A*β* in the hippocampal region leads to the formation of amyloid plaques, which inhibit neurogenesis and lead to cognitive dysfunction [17]. SD upregulates the expression of BACE1 and APP processing, which also play critical roles in AD [68]. SD increases cortisol level, a stress maker which was shown to impair cognition in AD patients [69]. Stress directly affects the brain in 3 different ways: (a) it enhances tau aggregation through hyperphosphorylation and causes genetic alterations in the DNA [71]; (b) it declines synaptic density and number of neurons and increase the deposition of A*β*; and (c) it also impairs cardiovascular, metabolic, and GI functions and weakens the immune system [70]. Chronic stress causes hypothalamic–pituitary–adrenal (HPA) axis imbalance, which leads to the accumulation of A*β* and tau proteins, cognitive impairment, and neuronal death, resulting in AD [71].

Aquaporin 4 (AQP4) is the most abundant water channel in the brain which regulates water homeostasis. AQP4 is highly expressed in astrocytes, lining of the ventricles, and in perivascular astrocytes end feet enclosed by blood vessels from the CNS [73, 74]. It also plays a crucial role in removing toxic metabolites including A*β* and tau and prevents their aggregation. SD impairs the glymphatic system which results in the aggregation of A*β* [17, 75]. A paravascular pathway facilitates CSF flow through the brain parenchyma and the clearance of interstitial solutes, including amyloid *β*. Studies report that glymphatic function declines over mid to terminal stages of AD due to impaired polarity of AQP4 at the astrocyte end feet [75, 76]. A recent report indicates a genetic mutation in AQP4 in patients with less sleep or SD [77].

Phosphorylated Tau proteins bind to the microtubule and provide structural stability to the neurons. Hyperphosphorylation of Tau is a hallmark of AD, which causes aggregation and formation of filamentous structures called Neurofibrillary tangles (NFT). Preclinical evidences reveal that an impaired sleep-wake cycle can increase the hyperphosphorylation of Tau proteins [78]. Apolipoprotein E (*APOE*) is one of the major constituent of chylomicrons and intermediate-density lipoproteins (IDLs) and is involved in regulating catabolism of lipoproteins [79]. *APOE* genes consist of three variants of genes -*ε*2, *ε*3, and *ε*4 [80]. *APOE/ε*4 variant gene is a risk factor of AD; *ε*4 allele also increases the deposition of A*β* and cognitive impairment [81]. Leoni et al. have shown that ApoE/*ε*4 is associated with sleep disorders and increases the levels of LDL and triglycerides. Brain removes excess cholesterol by converting it into polar 24-hydroxycholesterol (24-OHC) that is cleared through *APOE*. CSF samples from AD patients have shown an increase in the concentration of ApoE, tau, NFTs, and 24-OHC. Increased levels of these components lead to increased NFT loading cells [82]. It is suggested that ApoE/*ε*4 is less efficient in clearing cholesterol from the neurons and promotes NFT load in neurons. Lim et al. have also shown that adequate sleep inhibits the effects of APOE in the formation of NFT and delays the progression of AD [81].

Cholinergic neurons are essential in regulating sleep-wake states, memory, and learning [83]. Cholinergic neurons are active during REM sleep and wakefulness, and they are less active during NREM sleep [84]. SD increases adenosine levels in the basal forebrain, thereby inhibits the cholinergic system and disrupts the switch between sleep and wakefulness [85]. The stimulation of the muscarinic receptors enhances long-term potentiation and synaptic plasticity in the CA1 region of the hippocampus [86]. Experiments on animal models have also shown cognitive impairment following treatment with muscarinic antagonist [87]. BDNF expression is also regulated by the muscarinic receptors. These results suggest that equilibrium in cholinergic activity is essential for memory encoding and recall. Cognitive dysfunction, inability to learn new things, and difficulty in performing the known tasks all are the primary symptoms of AD. According to WHO, 30 million people across the globe are suffering from Alzheimer's dementia [88]. It is known that sleep is essential for cognition, and SD leads to memory deficits, impaired attention decision making, and retrieval [89]. REM sleep is essential for the LTP, whereas NREM sleep deprivation does not affect LTP [90]. SD increases the adenosine and cholinergic functions in various regions of the brain including the hippocampus and basal forebrain [91]. These results suggest the negative impact of SD on cholinergic neurons and cognitive functions.

Excitatory neurotransmission in the mammalian CNS is primarily regulated by the ionotropic glutamate receptors (iGLURs). These receptors play a crucial role in LTP, learning, and neuronal survival [92]. Abnormal signalling of these receptors is involved in various neurological disorders like Alzheimer's disease, Parkinson's disease, Huntington disease, and multiple sclerosis [93]. NMDA receptors, a division of iGLURs, due to its unique properties like voltage-dependent activation, enhanced Ca^2+^ influx and slow ligand-gated kinetics make them the key players in LTP [94, 95]. NMDA receptors are also crucial for neuronal survival. These receptors activate survival pathways like ERK, PI3K/AKT, and CaMK and also inhibit proapoptotic kinases like GSK3*β* [96]. Many studies suggest that overexcitation of glutaminergic neurons or excitotoxicity leads to neurodegeneration. This is due to an increased influx of Ca^2+^through the NMDA receptors [97–99]. Increased levels of Ca^2+^ influx lead to impaired synaptic function and eventually to neuronal cell death. This is closely associated with cognitive decline and pathological neural anatomy similar to AD patients.

**Figure 2:**
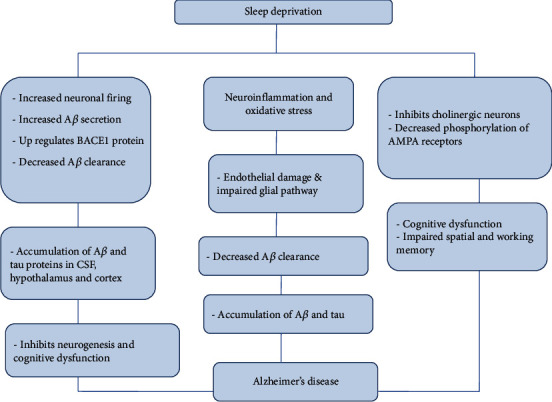
Sleep deprivation-induced alteration in various pathways leading to AD pathology. SD increases neuronal firing, upregulates BACE 1 proteins, and aggravates neuroinflammation and oxidative stress. Alterations in these pathways impair clearance of the toxic metabolites and leading to the accumulation of A*β* and tau proteins. SD has a negative impact on the cholinergic neurons, and this attributes to cognitive dysfunction and impaired memory in AD patients.

SD has a negative impact on LTP and synaptic plasticity and hippocampal glutaminergic NMDA receptors. SD reduces the expression of the GluN1 subunit of the NMDA receptors, thereby lowering the excitatory postsynaptic current in the CA1 region [100]. Studies also report that 12 h of SD leads to a drastic decrease in the phosphorylation of hippocampal AMPA receptors at the GluR1-S845 site and decreases the levels of AKAP150 scaffolding proteins [101]. All these data suggest that SD leads to impaired spatial and working memory by decreasing AMPA receptor phosphorylation through the decreasing levels of AKAP150 scaffolding proteins. Thus, SD is closely connected to the pathological markers and that it plays a crucial role in the pathogenesis of AD (Figure 2).

### 5.2. Parkinson's Disease

Parkinson's disease (PD) is a distinctive motor disease that involves neuromuscular rigidity, bradykinesia, and tremor. The accumulation of *α*-synuclein, A*β*, TDP-43, and tau is the major hallmarks of PD [102–105]. Sleep improved the cognitive and mental ability in PD patients [106]. Conversely, the destruction of nigrostriatal *dopaminergic neurons* or dorsal striatum has been reported to disrupt the sleep–wake cycle [107].

Paravascular clearance pathway-“Glymphatic system” is responsible for the clearance of the toxic metabolites from the brain [75, 104]. As noted in AD, the clearance of A*β* proteins through the glymphatic pathway and the clearance of *α*-synuclein are not reported as of today and need to be investigated. Hence, we propose that interventions promoting glymphatic clearance might be beneficial in PD. This could be a potential target at least in the early stages of the disease as it can prevent the accumulation of *α*-synuclein beyond mesencephalic and limbic regions.

Postmortem analysis of PD brains has shown to contain higher levels of oxidized proteins and lipids. SirT3 is a NAD-dependent deacetylase sirtuin-3 protein present in the inner mitochondrial membrane and is involved in ATP production and redox processes [108]. Metabolic homeostasis requires SirT3. Chronic SD impairs SirT3 activity that eventually leads to locus coeruleus neurons (LCns) superoxide production, mitochondrial protein acetylation, and neuronal death leading to PD [109]. PET imaging revealed that SD in healthy volunteers reduced the binding of raclopride (a radiotracer that binds to D2 and D3 receptors), which may be due to the downregulation of D2/D3 receptors in the ventral striatum or decreased receptor affinity [110]. The downregulation of D2/D3 receptors due to SD causes decreased wakefulness and other altered behavioural effects, which are mediated through the dopaminergic system. Thus, the downregulation of D2/D3 receptors may lead to PD-like symptoms [111]. On the contrary, Targa et al. (2018) reported that REM SD increases dopamine turnover in a rodent model of PD and potentiates dopaminergic activity [112]. SD in mouse models disrupts metabolic homeostasis in LCns, and chronic sleep deprivation (8 h) for three days disrupts the antioxidant defence system [109]. This leads to oxidative stress in LCns, in turn, causing a burst of reactive oxygen species (ROS) and superoxide (O^2-^) resulting in cholinergic damage in the basal forebrain and Pedunculopontine tegmental nucleus (PPT) in early PD state [113, 114]. These molecular, neurochemical, and imaging data demonstrate a close link between SD and PD.

### 5.3. Multiple Sclerosis

Multiple sclerosis (MS) is an immune-mediated neurodegenerative disease influenced by both genetic and environmental factors [115]. Fatigue is a common complaint in MS affecting 90% of the patients with low quality of life. Recent studies have shown that SD contributes to fatigue in MS [116]. Individuals working in shifts for at least three years before the age of 20 years have been found to be more susceptible to MS as compared to individuals working in day shifts [117]. A possible mechanism behind this increased risk is disruption in circadian rhythm; the release of cellular and molecular inflammatory mediators causes neuroimmune dysregulation [118]. SD affects the expression of genes involved in the synthesis and maintenance of the myelin proteins [119]. SD also downregulates the expression of genes involved in oligodendrocyte precursor cell (OPCs) differentiation, and these cells are necessary for the formation of new myelin components in both normal as well as injured brain [119]. SD is reported to upregulate the expression of apoptotic and cellular stress response genes which impedes nerve regeneration in MS. More studies are needed to clearly understand the link between SD and MS.

### 5.4. Huntington's Disease

Huntington's disease (HD) is a neurodegenerative disease caused by an extended polyglutamine tract in the huntingtin protein. It is characterized by an abnormal increase in CAG sequence in the gene that codes for huntingtin protein on chromosome 4 [120]. HD not only causes motor impairment, cognitive deterioration, and behavioural problems but it also disturbs sleep patterns. Pathological changes in hypothalamic suprachiasmatic nucleus molecular oscillation have been found to be involved with disturbances in the sleep-wake cycle in HD [121]. Disruptions in sleep lead to neuronal dysfunction and shrinkage in brain volume and immune dysregulation [122]. 95% of the striatal neurons are projection neurons like medium spiny neurons (MSN), which are GABAergic and inhibitory in nature [123]. MSNs play a crucial role in the striatal microcircuit, which is impaired in HD. MSN indirect pathway is adversely affected, and its projections like enkephalins are lost in HD [124]. Balanced discharge and firing of basal ganglia require appropriate REM sleep and wakefulness [125]. Also, SD adversely affects the cerebellar and basal ganglia loops [126], which may in turn lead to HD pathogenesis. SD impairs the network between the cerebellum and postcentral gyrus, which is located at the parietal lobe and primary somatosensory cortex and plays a key role on motor control [127, 128]. This reveals that SD worsens the motor functions in HD. Polysomnography of the patients with HD showed an increase in initiation time of sleep, fragmentation of sleep, frequent awakening, and reduced quality of sleep [128, 129]. SD not only impairs cognition but also causes depression in HD patients.

### 5.5. Sleep Deprivation and Stroke

Ischemic stroke models in rodents showed an increase in slow-wave sleep and inhibition of REM sleep [130, 131]. Chronic sleep restriction has been found to inhibit adult neurogenesis in the hippocampal region and aggravate neurodegeneration [132]. SD worsens cerebral injury following cardiac arrest in mice which results in the formation of glial scars [133]. Following ischemic stroke, there is a decrease in blood flow, excitotoxicity, reactive oxygen species (ROS) generation, and activation of inflammatory pathways [134, 135]. Also, SD increases brain temperature and glucose consumption which is confirmed by elevated 2-deoxyglucose uptake and decline in brain glycogen levels [136]. Local field potentials and EEG signals from the cortical region reveal an increase in glutamate levels following SD [137]. Increased glutamate levels aggravate the excitotoxicity induced by ischemia. SD decreases the antioxidant markers like SOD and GSH which alleviate the ROS burst in cerebral ischemia [138, 139].

A study in young men and women subjected to 40 h of total SD reported a significant increase in the plasma levels of inflammatory markers IL-1beta, IL-1ra, E-selectin, and ICAM-1 [140]. Since cerebral ischemia activates inflammatory pathways, SD during this period aggravates the inflammatory reactions. SD following cerebral ischemia increases the levels of growth-inhibiting gene Neurocan, a chondroitin sulfate proteoglycan released by astrocytes. Surrounding the infarct area densely packed astrocytes along with Neurocan forms a barrier and inhibits neuronal reconnection [131]. Normal sleep increases the levels of gamma-hydroxybutyrate (GABA) which has an inhibitory effect on ischemia-induced Neurocan [141]. This indicates that Neurocan levels are crucial in sleep-modulated poststroke brain plasticity. The exact mechanism of how sleep modulates brain plasticity following stroke warrants further studies. These investigations indicate that SD aggravates stroke pathophysiology by increasing the expression of growth-inhibiting genes, neuroinflammation, and oxidative stress.

### 5.6. Sleep Deprivation in Learning and Memory

SD has a negative impact on cognitive functions like attention, learning, memory formation, acquisition, and retrieval [57]. Following SD-impaired performance was observed in various cognitive tasks like Morris water maze, radial arm maze, and novel object recognition test [101, 142, 143]. The major effect of SD is that it impairs the ability of the brain to retain new information and impairs memory consolidation [57]. SD activates certain ion channels and causes synaptic alterations resulting in decreased membrane excitability in hippocampal CA1 neurons and inhibition of LTP production in CA1 neurons and dentate granules [144]. Thus, the membrane and synaptic regions play an important role in spatial memory deficits of SD rodents. SD also increases the spike frequency in the CA1 region, which in turn inhibits the encoding of spatial information in the hippocampus [145, 146].

Adverse effects of SD on LTP, synaptic plasticity, consolidation, retrieval, alterations in the intracellular signalling molecule, and receptors like NMDA are discussed in the above sections; apart from this, SD also has a negative impact on cognition related signalling molecules as discussed below.

#### 5.6.1. Calcium Calmodulin-Dependent Protein Kinase II (CaMKII)

CaMKII is important for LTP in the hippocampus [147]. SD adversely affects intracellular signalling and downregulates basal and phosphorylated CaMKII in CA1 and dentate gyrus [148]. The level of calcineurin is shown to increase following SD. This leads to the dephosphorylation of CaMKII, thereby, reducing the levels of pCaMKII during E-LTP [148]. Phosphorylation of CaMKII at Thr286 is essential for NMDA receptor-dependent LTP in the hippocampal CA1 region [149]. SD for 24 h shows a drastic decrease in the ratio of CaMKII to pCaMKII. These data suggest that SD impairs the phosphorylation of CaMKII [143, 148].

#### 5.6.2. Calcineurin

Calcineurin is also known as Protein Phosphatase 2B and is involved in the regulation of Ca^2+^ and calmodulin. Ca^2+-^calmodulin complex activates calcineurin which triggers the release of Protein Phosphates 1 (PP1), which inactivates CaMKII [150, 151]. Higher concentrations of Ca^2+^- CaMKII complex is required to induce and maintain LTP [152]. Increased calcineurin levels impair LTP in the hippocampal CA1 region [153]. 72 h of SD has been found to increase calcineurin levels in the hippocampal region [154]. The inhibition of calcineurin promotes LTP in the hippocampus [155]. In contrast, some studies have also shown that calcineurin levels are not altered by SD [156].

#### 5.6.3. cAMP Response Element Binding Protein (CREB)

SD impairs translation and causes a negative impact on genes which are essential for memory formation, consolidation, and retrieval [113, 157, 158]. Transgenic animal model has revealed that proteins like cAMP response element-binding protein (CREB) are essential for memory consolidation and long-term synaptic plasticity. CREB is a downstream messenger in the cAMP-PKA pathway [159]. cAMP signalling is maximum during the REM sleep [160], and SD inhibits cAMP signalling [144]. Molecular studies have also revealed that SD potentiates the activity of cAMP degrading phosphodiesterases (PDEs) and impairs cAMP signalling and produces a negative effect on memory and long-term potentiation [144]. SD not only impairs spatial and working memory but also affects long-term memory and decision-making. SD disrupts the memory consolidation period and impairs memory [161].

#### 5.6.4. Brain-Derived Neurotrophic Factor (BDNF)

BDNF is crucial for the growth and survival of neuronal cells, neurotransmission, LTP, learning, and memory. Binding of BDNF to tyrosine kinase receptors activates signalling cascades leading to the production of CREB and CBP, which are very important transcription factors and important for normal functioning and development of the brain [162]. BDNF gene expression was seen to increase following learning and LTP induction [163, 164]. BDNF activates the release of Ca^2+^ through tropomyosin kinase-B (TrkB) receptor which in turn activates CaMKII and CREB [165–167]. SD reduces BDNF levels in the hippocampal CA1 and DG regions [167]. SD also leads to the suppression of BDNF upregulation, thereby decreasing extracellular BDNF availability [143, 148, 168].

### 5.7. Epilepsy

Interrelation between epilepsy and sleep is established for long. 20% of seizures and increased epileptiform in epilepsy patients occurs during sleep [169]. Epileptiform discharges occur during NREM sleep in a synchronized pattern, while desynchronized discharges occur in REM sleep [170]. Pittsburgh Sleep Quality Questionnaire (PSQI) study, 16.9%–28% of the epileptic patients suffer from daytime sleepiness, and 24.6%–34% of patients suffer from insomnia. Different forms of epilepsy cause various types of sleep-related problems, for example, temporal lobe epilepsy causes sleep disturbances [171, 172], while frontal lobe epilepsy causes sleep fragmentation and daytime sleepiness [173]. Also, patients with partial seizures have more sleep-related problems than patients that have generalized seizures [174]. A series of studies conducted during 1960-1970 reported that SD initiates epileptic seizures and facilitate epileptiform discharges [171]. Studies have also shown that patients with generalized seizures who have undergone SD are prone to epileptic seizures [175]. Reports indicate that army officers and pilots who are sleep deprived and people who are chronic alcohol consumers show generalized tonic-clonic seizures [176]. Electroencephalogram recordings have shown that SD increases interictal epileptiform discharges (IEDs) [177, 178]. However, the pathophysiological mechanisms involving this are still not clear. Also, SD affects behavioural and membrane excitability. Rats subjected to 72 h SD show poor performance in behavioural tasks involving the hippocampus and amygdala and show reduced membrane excitability [179]. It was also found that CA1 neurons had a lower membrane output resistance and low action potential with respect to depolarizing current, which leads to the initiation of seizures. EEG studies of sleep-deprived patients and studies involving drug-induced sleep have conflicting results. Some studies reported that drug-induced sleep shows a greater epileptiform discharge [180], while others report greater epileptiform discharge in sleep-deprived patients [180, 181]. SD also has an impact on the onset as well as the clinical signs of the seizures. The risk of seizure onset is reported after 48 h of SD [182, 183]. A study reported that of 100 awakening epileptic patients, 65 % of the seizures were due to sleep deprivation [184].

Antiepileptic drugs potentially influence sleep. Phenytoin decreases the onset of sleep, Phenobarbital reduces onset and arousal from sleep, and gabapentin also has similar effects. An increased NREM sleep was seen with pregabalin and carbamazepine [185]. In contrast, levetiracetam decreased NREM sleep. Ethosuximide and gabapentin increased REM sleep. Day time sleeping was reported to be induced because of intake of topiramate, lamotrigine, and zonisamide [185]. The effect of antiepileptic drugs on sleep varies between individuals. Hence, there is a need for a careful patient monitoring system. Further studies are also needed in this context to improve night sleep in epileptic patients and to understand how SD influences seizures.

### 5.8. Autism Spectrum Disorders

Autism spectrum disorder (ASD) comprises of autism and Asperger's syndrome and involves severe neurodevelopmental disorder. It affects all phases of child development and involves symptoms such as inability to communicate, lack of socialization, and behavioural problems [186]. According to World Health Organization (WHO), one in 160 children suffers from ASD. Symptoms are usually observed during early age, combined with amnesia, sleep disorders, and learning disabilities [187]. National Sleep Foundation has recommended sleep research involving ASD children as a high priority [188]. This is because the pathophysiology and neurochemistry of ASD puts the children in severe stress and sleep disturbances [189]. Polysomnography studies show that ASD children have shorter sleep duration, low sleep quality, and low REM sleep [187]. Limoges et al. (2005) have reported fewer spindles of stage 2 sleep in ASD patients when compared to normal patients [190]. Sleep disturbances are associated with an increase in the severity of autism scores [191] and involve impaired social interaction [192].

The main cause of sleep-related problems in ASD is impaired melatonin secretion, impaired circadian clock, and genetic mutations [190, 193]. Onset of sleep problems, involving early waking, and poor quality of sleep may be due to late peaking in melatonin concentration. In a randomized controlled trial, ASD children supplemented with melatonin were found to have reduced onset of sleep time, reduced number of night waking, and improved in the quality and duration of sleep [194]. Mutation in circadian clock gene Per1 and Npas2 is found in 110 ASD patients [195]. This evidence suggests that sleep deprivation or sleep-related problems affect the daily functioning of ASD patients.

### 5.9. Glioma

Glioma is the most common primary brain tumour and involves astrocytes, ependymal, and oligodendritic cells [196–198]. 30-50% of glioma patients suffer from symptoms of insomnia [199]. WHO has characterized glioma into four categories:

- Grade 1: tumours that can be detected early
- Grade 2: tumour with hypercellularity
- Grade 3: astrocytoma and
- Grade 4: glioblastoma

Sleep has been reported to play a vital role in immunity and homeostasis [200, 201]. Sleep disturbances have a severe adverse impact on quality of life. The genetic basis of sleep regulation is well understood, and data suggests that sleep disturbances may lead to mutations in genes [202]. Proinflammatory Cytokines IL1*β*, IL2, and NF*κ*B2 are critically involved in sleep-wake disturbances in cancer patients [203]. Sleep disturbances in brain tumour patients may be due to the tumour or effect of medication. Somnolence syndrome is seen in glioma patients following cranial radiation therapy, and the symptoms include fatigue, drowsiness, and in coordination. Reports also reveal Hypothalamic–pituitary–adrenal axis dysregulation and impaired melatonin and hypocretin secretion. Melatonin possesses potent anti-inflammatory activity as it alleviates various cytokines like IL1*β*, IL2, IL6, and TNF*α* [204]. Melatonin also inhibits prostaglandin [205] and COX2 enzymes [206]. It has also been reported that MT3 receptors express QR2, which is a phase 2 detoxification enzyme. Melatonin acts as a cosubstrate and enhances the production of QR2, thus suppressing tumour initiation and progression [207]. Melatonin also shows affinity towards orphan receptors RZR/ROR and modifies transcription of genes involved in cellular proliferation and translation of proteins like 5-lipoxygenase and bone sialoprotein. It also enhances the expression of P53, a tumour suppressor gene [208]. Furthermore, melatonin inhibits VEGF which is a heparin-binding glycoprotein and stimulates angiogenesis. Thus, the sleep-promoting hormone melatonin also helps in stopping tumour progression [209]. These data suggest a possible link between sleep/SD and cancer.

Orexin, a neuropeptide, has an important role in sleep-wakefulness and appetite [210]. Orexin receptors OX1 and OX2 are found in C6 glioma cells. Orexin A shows a strong affinity towards OX1 receptors in comparison to orexin B. However, both show a similar affinity towards OX2. Orexin A decreases the survival of C6 glioma cell lines. The possible mechanism through which orexin decreases survival is (a) orexin stimulates Caspase 3 activity leading to apoptosis and (b) inhibition of cell survival [210]. Thus, orexin plays a vital role in controlling and eliminating glioma. SD or alterations in sleep-wake cycle have been shown to change orexin levels and indirectly impact glioma to some extent.

### 5.10. Neuropathic Pain

Neuropathic pain is caused due to nerve injury while central neuropathic pain arises due to stroke [211], MS [212], and spinal cord injury [213]. 25% of diabetic and 35% of HIV patients suffer from neuropathy pain [214]. In a healthy population, SD increases pain sensitivity and intensity [215]. It was seen that rats subjected to 72 h of SD showed an increase in pain response against noxious mechanical and electrical stimuli [216]. SD increases glutamate concentration [217] and also affects the opioid system [218] stimulating the nociceptive system. In humans and rodents, SD causes increased WBC counts and enhances the level of C-reactive protein, IL1*β*, IL6, and TNF*α* [219–221]. SD stimulates astrocytic phagocytosis which leads to microglial activation in the cortical region of mice [221]. Total sleep deprivation following chronic constriction injury (CCI) increases microglial activation in the cuneate nucleus (CN) and exasperates neuropathic pain induced by nerve injury. Melatonin administration reduces the level of proinflammatory cytokines, thereby reducing neuropathic pain in CCI rats [222]. Melatonin blocks Ca^2+^ channels directly by binding to the receptors (MT1 and MT2) or indirectly by binding to G protein-coupled receptors [223, 224]. Recently, we reviewed the role of body fluids on sleep [225]. Blocking of Ca^2+^ channels leads to a decrease in glutamate concentration and results in the suppression of NMDA receptors and pain.

## 6. Conclusion

**Figure 3:**
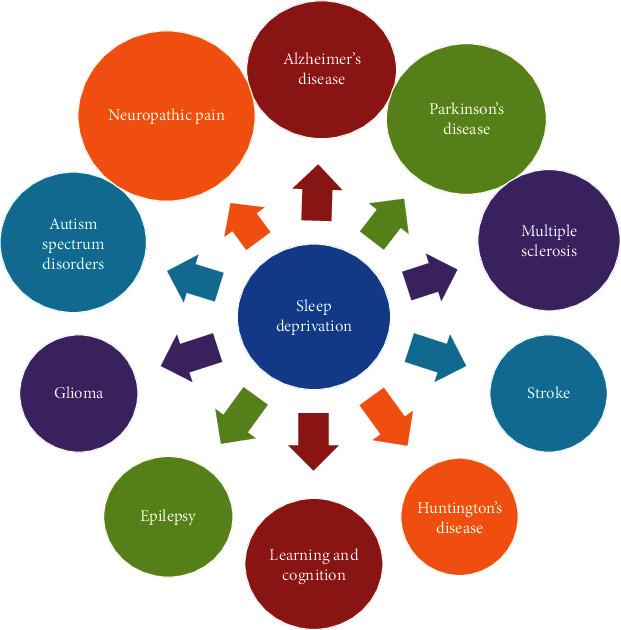
Sleep is a vital phenomenon and an indicator of overall health. Normal sleep is very essential for memory and brain health since various neural circuits in the brain are involved in sleep. Sleep deprivation has evolved as a major threat in modern society. SD impairs LTP and molecules associated with memory and leads to cognitive dysfunction. SD also impairs the clearance of toxic metabolites produced in the brain and contributes to the pathophysiology of neurological disorders like AD, PD, and cerebral stroke. SD also causes an imbalance in the immune system and aggravates the pathophysiology of MS and glioma. It can be concluded that SD adversely affects various proteins, genes, and molecular cascades in neurodegenerative disorders.

Based on the data from the American Sleep Apnea Association, “It is estimated that sleep-related problems affect 50 to 70 million Americans of all ages and socioeconomic classes.” Since one-third of an average person's life is spent in sleeping; sleep is an important aspect that influences human function and performance during the remaining two-thirds. Thus, it is important to understand the effect of sleep loss and sleep disorders on human health. Since the brain governs various sleep patterns, SD is one of the reasons for the etiopathogenesis of various neurological disorders. Sleep deprivation impairs LTP and brain-derived neurotrophic factors and is linked to dementia and cognitive decline. SD also causes accumulation or misfolding of proteins and its role in neurodegenerative diseases like AD, PD, and cerebral stroke is well documented. SD is associated with an imbalance in the immune axis leading to increased release of cytokines, autoimmune diseases (multiple sclerosis), and glioma (Figure 3). In summary, it is apparent that SD plays a role in adversely modulating several key proteins and cascades cellular/molecular levels in various neurological disorders. Based on the data gathered from literature and clinical studies on SD, it is apparent that the impact of SD is enormous and profound and needs urgent attention. Furthermore, it might not be an overstatement to say that—if left unattended and overlooked, SD will impose severe healthcare and medical economic burden in the society.

## Abbreviations

AD: Alzheimer's disease
AMPK: AMP-activated protein kinase
APOE: Apolipoprotein E
AQP4: Aquaporin-4
ASD: Autism spectrum disorder
A*β*: Amyloid beta
BDNF: Brain-derived neurotrophic factor
BF: Basal forebrain
CaMKII: Calcium calmodulin-dependent protein kinase II
cAMP: Cyclic AMP
CCI: Chronic constriction injury
CREB: cAMP response element-binding protein
CRP: C-reactive protein
dDpMe: Dorsal deep mesencephalic reticular nucleus
DG: Dentate gyrus
DPGi: Dorsal paragigantocellular reticular
EEG: Electroencephalogram
GABA: Gamma-aminobutyric acid
GiA: Alpha gigantocellular reticular
GiV: Gigantocellular reticular
GluN1: Glutamate ionotropic receptor NMDA type subunit 1
GSH: Glutathione
HD: Huntington's disease
IDLs: Intermediate density lipoproteins
IL-6: Interleukin 6
IPSP: Inhibitory postsynaptic potential
ISF: Interstitial fluid
LH: Lateral hypothalamic
LPGi: Lateral paragigantocellular
LTP: Long-term potentiation
MCH: Melanin-concentrating hormone
MnPO: Median preoptic nucleus
MS: Multiple sclerosis
MSN: Medium spiny neurons
NFT: Neurofibrillary tangles
nNOS: Neuronal nitric oxide synthase
NO: Nitric oxide
NREM: Nonrapid eye movement
OPC: Oligodendrocyte precursor cells
PD: Parkinson's disease
PKA: Protein kinase A
POA: Preoptic area
PP1: Protein phosphates 1
PSQI: Pittsburgh Sleep Quality Questionnaire
REM: Rapid eye movement
ROS: Reactive oxygen species
SCN: Suprachiasmatic nucleus
SLD: Sublaterodorsal tegmental nucleus
SOD: Superoxide dismutase
TNF-*α*: Tumour necrosis factor
VLPO: Ventrolateral preoptic nucleus

## Supplementary Materials

Supplementary materials Supplementary Table 1: genes detected as differentially regulated in sleep deprivation microarray (multiple testing corrected *P* value < 0.05) tested by qPCR. All comparisons between sleep-deprived (SD) and nonsleep-deprived (NSD) samples measured by qPCR are significant at *P* < 0.05 using 1-tailed *t*-tests, except Nr4a1. For each gene, qPCR expression is represented as the fold change (FC) in SD mice relative to NSD mice, normalized to the average expression of the housekeeping genesActg, Hprt, and Tuba4a (used with permission). Supplementary Figure 1: quantitative RT-PCR validation of genes upregulated or downregulated by sleep deprivation in the hippocampus. Quantitative RT-PCR (dark gray) was used to validate the expression level of genes identified by microarray analysis (light gray) as being changed in the hippocampus by sleep deprivation. For each gene, the expression is represented as the fold change in SD mice relative to NSD mice, normalized to the average expression of housekeeping genes Actg, Hprt, andTuba4a. The fold change values from the microarray for SD/NSD are shown for each gene for comparison. All SD/NSD qPCR comparisons are significant at *P* < 0.05, except Nr4a1 induction (see Table 1). Bars indicate ±SE (used with permission).
