# Supplementary material for: Sleep Deprivation and Neurological Disorders
Source: Biomed Res Int. 2020 Nov 23;2020:5764017. doi: 10.1155/2020/5764017 (PMC7755475; doi:10.1155/2020/5764017)
Supplement: Supplementary materials — Supplementary Table 1: genes detected as differentially regulated in sleep deprivation microarray (multiple testing corrected P value < 0.05) tested by qPCR. All comparisons between sleep-deprived (SD) and nonsleep-deprived (NSD) samples measured by qPCR are significant at P < 0.05 using 1-tailed t-tests, except Nr4a1. For each gene, qPCR expression is represented as the fold change (FC) in SD mice relative to NSD mice, normalized to the average expression of the housekeeping genesActg, Hprt, and Tuba4a (used with permission). Supplementary Figure 1: quantitative RT-PCR validation of genes upregulated or downregulated by sleep deprivation in the hippocampus. Quantitative RT-PCR (dark gray) was used to validate the expression level of genes identified by microarray analysis (light gray) as being changed in the hippocampus by sleep deprivation. For each gene, the expression is represented as the fold change in SD mice relative to NSD mice, normalized to the average expression of housekeeping genes Actg, Hprt, andTuba4a. The fold change values from the microarray for SD/NSD are shown for each gene for comparison. All SD/NSD qPCR comparisons are significant at P < 0.05, except Nr4a1 induction (see Table 1). Bars indicate ±SE (used with permission). [file 5764017.f1.pdf]

Supplementary Table 1: Genes detected as differentially regulated in sleep deprivation microarray (multiple testing corrected P value <0.05) tested by qPCR. All comparisonsbetween sleep-deprived (SD) and non-sleep-deprived (NSD) samples measured by qPCR are significant at P< 0.05 using 1-tailed t-tests, except Nr4a1. For eachgene, qPCR expression is represented as the fold change (FC) in SD mice relative to NSD mice, normalized to the average expression of the housekeeping genesActg, Hprt, and Tuba4a (Used with permission)

Gene Name	Probe-set ID	Microarray FC	qPCR FC	qPCR P Value	SD, *n*	NSD, *n*	Forward Primer	Reverse Primer
Fos	1423100_at	2.24	1.46	2E-03	9	9	CCGACTCCTTCTCCAGCA	TGTCACCGTGGGGATAAAGT
Arc	1418687_at	1.87	1.38	8E-03	8	9	AGCAGCAGACCTGACATCCT	GGTGTCATTCTCCTGGCTCT
Hspb1	1425964_x_at	1.77	2.07	1E-04	10	9	CACAGTGAAGACCAAGGAAGG	GGTGAAGCACCGAGAGATGT
Adamts2	1455720_at	1.61	1.38	3E-02	8	9	GAACCACAGGCTCTGGACAC	AACTGGACCACCGAGTCATC
Hspa8	1431182_at	1.57	1.18	2E-03	9	8	CGGAAGAGCTGATGGTAAAGA	CACCTTCCTGCTGCTTCTCT
Tsc22d3	1420772_a_at	1.56	1.67	1E-06	19	19	TTCTCTGCTTGGAGGGGATT	GCTCACGAATCTGCTCCTTT
Hspa5	1416064_a_at	1.50	1.66	1E-05	10	9	GGAGACTGCTGAGGCGTATT	TGGGCATCATTGAAGTAAGC
Nr4a1	1416505_at	1.43	1.08	1E-01	9	9	AAAATCCCTGGCTTCATTGAG	TTTAGATCGGTATGCCAGGCG
Prkab2	1435874_at	1.39	1.39	3E-04	10	10	GGGAAAGGAGCACAAGATC	CTGCTGCCAGGGTACAAAC
Htr1a	1450219_at	1.39	1.20	5E-02	9	9	CTGTTTATCGCCCTGGATGT	ATGAGCCAAGTGAGCGAGAT
Lats2	1419678_at	1.39	1.23	3E-02	14	13	ACCAAATTACATCGCTCCGG	TCGTCTCCCAGTTGATCACCT
Elk1	1421897_at	1.38	1.35	2E-03	15	15	TCAAGTTGGTGGATGCAGAG	GCAACCTCTGGGTAGGACAC
Prkaa2	1434766_at	0.79	0.88	5E-02	10	10	GGAATCCCGAAGCCAGAGCAA	CGATGCTTTTGAAGTCCAGA
Prkab1	1424119_at	0.79	0.81	3E-02	10	10	CATCCTCCCGCCACACCTGC	GAGCACCATCACTCCATC
Kcnv1	1439333_at	0.78	0.79	8E-03	8	9	GTCTGGTCCAGGTCTTGAGG	CACCATGCACAAGGAACACT
Hnrpdl	1449039_a_at	0.72	0.75	3E-03	13	14	AAGAACCAGCAGGATGACGGT	TGCAGTCTACCACTTCCCCAA
Usp2	1417168_a_at	0.66	0.82	7E-03	8	9	AAAGAGTGCCCAGGGTCTG	TCTCTCAGCTCTCGGGTGTT
Sirt7	1457279_at	0.65	0.84	3E-02	10	10	GAGCCTACCCTCACCCACA	GTGGAGCCCATCACAGTTC
Rbm3	1429169_at	0.54	0.72	2E-02	14	13	AGGACTTGCCTTCTGCCATGT	ATAGGCCCAAAGCTGCTGAA

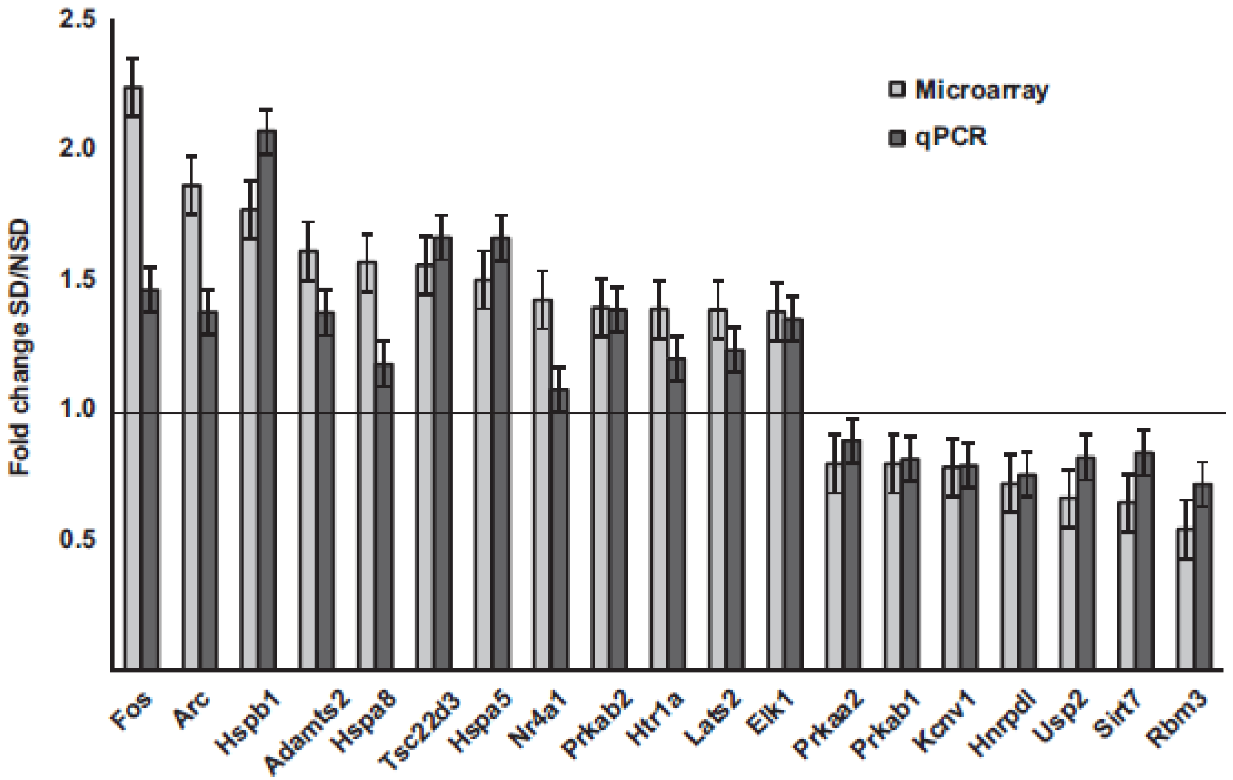

Supplementary Fig.1. Quantitative RT-PCR validation of genes upregulatedor downregulated by sleep deprivation in thehippocampus. Quantitative RT-PCR (dark gray) wasused to validate the expression level of genes identifiedby microarray analysis (light gray) as being changed inthe hippocampus by sleep deprivation. For each gene,expression is represented as the fold change in SD micerelative to NSD mice, normalized to the average expressionof housekeeping genes Actg, Hprt, andTuba4a. The fold change values from the microarrayfor SD/NSD are shown for each gene for comparison.All SD/NSD qPCR comparisons are significant at $P < 0.05$, except Nr4a1 induction (see Table 1). Bars indicate ± SE.(Used with permission)
